# Potential of Cry10Aa and Cyt2Ba, Two Minority δ-endotoxins Produced by *Bacillus thuringiensis* ser. *israelensis*, for the Control of *Aedes aegypti* Larvae

**DOI:** 10.3390/toxins12060355

**Published:** 2020-05-29

**Authors:** Daniel Valtierra-de-Luis, Maite Villanueva, Liliana Lai, Trevor Williams, Primitivo Caballero

**Affiliations:** 1 Departamento de Agronomía, Biotecnología y Alimentación, Universidad Pública de Navarra, 31006 Pamplona, Spain; daniel.valtierra@unavarra.es (D.V.-d.-L.); maite.villanueva@unavarra.es (M.V.); liliana.lai@unavarra.es (L.L.); 2 Bioinsectis SL, Avda Pamplona 123, 31192 Mutilva, Spain; 3 Instituto de Ecología AC, 91073 Xalapa, Mexico; trevor.inecol@gmail.com; 4 Institute for Multidisciplinary Applied Biology Research (IMAB), Universidad Pública de Navarra, 31006 Mutilva, Spain

**Keywords:** *Bacillus thuringiensis*, *Aedes aegypti*, minor proteins, synergy, mosquito control, Bti

## Abstract

*Bacillus thuringiensis* ser. *israelensis* (Bti) has been widely used as microbial larvicide for the control of many species of mosquitoes and blackflies. The larvicidal activity of Bti resides in Cry and Cyt δ-endotoxins present in the parasporal crystal of this pathogen. The insecticidal activity of the crystal is higher than the activities of the individual toxins, which is likely due to synergistic interactions among the crystal component proteins, particularly those involving Cyt1Aa. In the present study, Cry10Aa and Cyt2Ba were cloned from the commercial larvicide VectoBac-12AS^®^ and expressed in the acrystalliferous Bt strain BMB171 under the *cyt1Aa* strong promoter of the pSTAB vector. The LC_50_ values for *Aedes aegypti* second instar larvae estimated at 24 hpi for these two recombinant proteins (Cry10Aa and Cyt2Ba) were 299.62 and 279.37 ng/mL, respectively. Remarkable synergistic mosquitocidal activity was observed between Cry10Aa and Cyt2Ba (synergistic potentiation of 68.6-fold) when spore + crystal preparations, comprising a mixture of both recombinant strains in equal relative concentrations, were ingested by *A. aegypti* larvae. This synergistic activity is among the most powerful described so far with Bt toxins and is comparable to that reported for Cyt1A when interacting with Cry4Aa, Cry4Ba or Cry11Aa. Synergistic mosquitocidal activity was also observed between the recombinant proteins Cyt2Ba and Cry4Aa, but in this case, the synergistic potentiation was 4.6-fold. In conclusion, although Cry10Aa and Cyt2Ba are rarely detectable or appear as minor components in the crystals of Bti strains, they represent toxicity factors with a high potential for the control of mosquito populations.

## 1. Introduction

*Bacillus thuringiensis* ser. *israelensis* (Bti) was the first Bt serotype found to be toxic for dipteran species [1]. Bti forms parasporal inclusion bodies composed of insecticidal proteins (δ-endotoxins) that are widely used as the basis for microbial larvicides against dipteran species of medical importance, including mosquitoes, blackflies and chironomids [2,3]. Bti based products are considered to be powerful and highly selective larvicides for the control of disease vectors [4,5,6]. Indeed, Bti has been used to control mosquitoes for more than 35 years with almost no resistance report in vector populations [7,8]. The absence of resistance is likely due to the different modes of action and the synergistic effects of the multiple crystal proteins present in Bti-based products [9,10,11].

The parasporal crystal of Bti contains large amounts of four toxins: Cry4A, Cry4B, Cry11A and Cyt1A [12]. In addition, Cry10Aa and Cyt2Ba have also been described in some Bti strains, although these are expressed and accumulate in the crystal in much smaller quantities that the four main components [13,14]. All six of these proteins are encoded in the fully sequenced Bti plasmid pBtoxis [15]. The Cry10Aa protein was cloned and named CryIVC, according to the existing classification at that time, but was described as a protein with a low larvicidal potency against *A. aegypti* (Diptera; Culicidae) [16]. Later, the *cry10Aa* gene was identified as part of an operon that comprises two open reading frames (*orf1* and *orf2*) separated by a 66 bp gap [15]. Cloning of the complete operon, linked to the strong promoter of the *cyt1A* gene, revealed that Cry10Aa was expressed at high levels and exhibited high larvicidal activity, both alone and in combination with Cyt1A [17]. In contrast, although present at relatively low abundance in the Bti crystal [18], Cyt2Ba exhibited activity against *A. aegypti* larvae, but lower than the better-studied Cyt1Aa protein [19]. 

The interactions among the Cry and Cyt proteins of Bti have received more attention than any of the other Bt serovars [20,21,22,23,24]. Interactions involving the Cyt1A protein have attracted particular attention given the capacity of this protein to enhance the insecticidal activity of Cry proteins in strains of Bti [9,17,20,24], and those of Bt strains belonging to other subspecies [25]. Conversely, studies on the interactions of Cyt2Ba with other components of the Bti crystal are restricted to a single report of low synergistic activity of Cyt2Ba with the Cry4Aa protein [21].

The objective of this study was to quantify the larvicidal activity of the δ-endotoxins Cry10A and Cyt2Ba, which are minor components of the parasporal crystal of some Bti strains, against *A. aegypti*. To produce high amounts of these minority proteins, two recombinant Bt strains were constructed. One of these recombinants produced a crystal whose only component were the two Cry10Aa proteins, while the other only produced Cyt2Ba. We provide evidence that these proteins interacted synergistically to a remarkable degree when simultaneously ingested by *A. aegypti* larvae. 

## 2. Results

### 2.1. Insecticidal Cry and Cyt Genes Identified in Bti Strain from VectoBac-12AS^®^

A bioinformatic analysis of the genome of the Bti strain isolated from the commercial product VectoBac-12AS^®^, revealed that this strain contains a complex of insecticidal genes, including *cry* genes (*cry4Aa*, *cry4Ba*, *cry10Aa*, *cry11Aa*, and *cry60Aa*/*cry60Ba*) and *cyt* genes (*cyt1Aa*, *cyt2Ba*, and *cyt1Ca*). Unfortunately, it was not possible to obtain the complete sequence of the *cry4Aa* and *cry4Ba* genes, because they appeared distributed in various contigs. The rest of the *cry* genes shared 100% identity and similarity with some of the gene variants that have been previously described. Thus, *cry10Aa* was completely identical to *cry10Aa3* [15], *cry11Aa* was identical to *cry11Aa1* [26], and *cry60Aa*/*cry60Ba* were identical to *cry60Aa2*/*cry60Ba2* [27]. The three *cyt* genes (*cyt1Aa*, *cyt2Ba*, and *cyt1Ca*) identified in the VectoBac-12AS^®^ strain were also fully identical to *cyt1Aa1* [28], *cyt2Ba1* [18], and *cyt1Ca1* [15], respectively. 

### 2.2. Cloning of Cyt2Ba, Cry10Aa and Cry11Aa

The pairs of primers designed for *cyt2Ba, cry10Aa* and *cry11Aa* amplified fragments of 1536, 3813 and 2634 bp, respectively. The *cry10A* cloned fragment contained two open reading frames (*orf1* and *orf2*) in the nucleotide sequence, codifying for proteins of 680 and 489 amino acids, respectively. The *cry11Aa* amplicon encoded a protein of 646 amino acids but it also contained the *p19* gene located before *cry11Aa,* in line with the usual order of genes in this operon. The amplicon was cloned in a pSTAB plasmid containing the *p20* chaperon, which improves Cry11Aa synthesis and crystal formation [29]. The amplified fragment of *cyt2Ba* contained a single ORF which codified for a protein of 263 residues. The *cry4A* and *cry4Ba* genes, previously described and cloned by Delecluse et al. [30], were used in this study. 

### 2.3. Characterization of Bt Recombinant Strains Expressing cyt2Ba, cry10Aa, cry4Aa, cry4Ba and cry11Aa

Daily microscopical observation of the growth of the recombinant Bt strains in CCY medium confirmed that all of them produced spores and crystals between 36 and 48 h after the medium was inoculated. As expected, vegetative cells of BMB171 strain transformed with an empty plasmid produced endospores but no crystals. 

**Figure 1:**
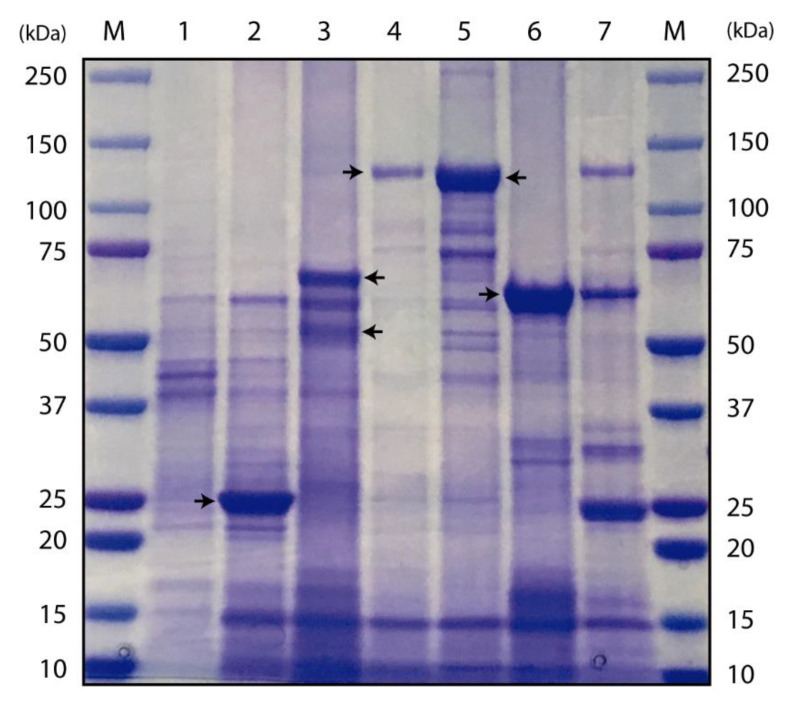
SDS-PAGE gel showing the protein profiles of the recombinant Bt strains and the strain present in VectoBac-12AS^®^. Lane M, molecular mass marker; lane 1, BMB171 acrystalliferous strain with an empty plasmid; lane 2, BMB171-Cyt2Ba; lane 3, BMB171-Cry10Aa; lane 4, 4Q2-81-Cry4Aa; lane5, 4Q2-81-Cry4Ba; lane 6, BMB171-Cry11Aa; lane 7, wild-type Bti strain from VectoBac-12AS^®^. Arrows indicate major protein bands.

SDS-PAGE showed that the recombinant BMB171-Cry10Aa expressed two proteins with molecular masses of approximately 68 and 56 kDa, which corresponded to the predicted sizes of the proteins encoded by *orf1* and *orf2*, respectively, of the *cry10Aa* operon (Figure 1, lane 3). Samples of spores and crystals from the rest of the recombinant strains (BMB171-Cyt2Ba, 4Q2-81-Cry4Aa, 4Q2-81-Cry4Ba and BMB171-Cry11Aa) generated characteristic major bands of approximately 29, 134, 128, and 73 kDa, respectively (Figure 1). The electrophoretic mobility of all these bands correlated well with the molecular mass of the proteins Cyt2Ba (lane 2), Cry4Aa (lane 4), Cry4Ba (lane 5) and Cry11Aa (lane 6). 

### 2.4. Mosquitocidal Activity of the δ-Endotoxins Produced by Bti

Single-concentration bioassays involving an estimated LC_30_ concentration of inoculum in all cases were performed on mixtures of Cyt2Ba with each of the Cry4Aa, Cry4Ba, Cry10Aa and Cry11Aa proteins. The results of these assays indicated that *A. aegypti* second instar larvae treated with combinations of Cry10Aa+Cyt2Ba and Cry4Aa+Cyt2Ba experienced high mortality compared to the mortality values observed in insects treated with each of the toxins separately (Table 1). In contrast, no evidence of potentiation of larval mortality was observed for mixtures of Cry4Ba+Cyt2Ba or Cry11Aa+Cyt2Ba. For this reason, the 1:1 mixtures of Cry10Aa+Cyt2Ba and of Cry4Aa+Cyt2Ba were selected for subsequent concentration-mortality studies.

Table 2 shows the raw mortality data of a series of concentrations for Cry10Aa, Cyt2Ba and the combination of both. Analogously, Table 3 shows the raw mortality data of a series of concentrations for Cry4Aa, Cyt2Ba and the combination of both.

**Figure 2:**
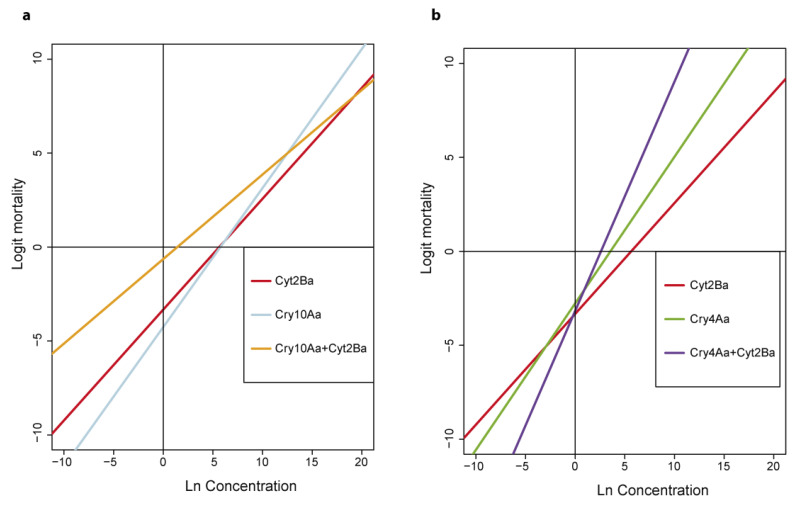
Graphical representation of the logit regression lines for the individual toxins and the toxin combinations. (**a**) Regression lines for Cyt2Ba, Cry10Aa and Cry10Aa+Cyt2Ba. (**b**) Regression lines for Cyt2Ba, Cry4Aa and Cry4Aa+Cyt2Ba.

Regression lines were performed for the individual toxins and the mixture of toxins (Figure 2) which were then used to estimate median lethal concentrations (LC_50_) (Table 4).

Recombinant Cry10Aa and Cyt2Ba proteins exhibited a high insecticidal activity against *A. aegypti* second instar larvae when inoculated individually. The LC_50_ values estimated for Cry10Aa and Cyt2Ba were 299.62 ng/mL and 279.37 ng/mL, respectively. The VectoBac-12AS^®^ wild-type strain, incorporated into the bioassays as a positive control, had an LC_50_ value of 1.02 × 10^−1^ ng/mL and the BMB171 strain with the empty plasmid resulted in no mortality (Table 4). The slopes of the regression lines corresponding to Cry10Aa and Cyt2Ba did not differ significantly (F_1,8_ = 0.620, *p* = 0.454), whereas the slope of the mixture of Cry10Aa+Cyt2Ba was significantly lower than that of the individual toxins (F_2,12_ = 7.359, *p* = 0.008). The observed LC_50_ value for Cry10Aa+Cyt2Ba was 4.22 ng/mL whereas the expected LC_50_ value was 289.27 ng/mL, assuming additive action of each of the toxins [31]. The estimated potentiation of Cyt2Ba and Cry10Aa proteins when ingested together and in the same relative proportions, was 68.6-fold (Table 4). 

In contrast, the slopes of the regressions of the individual Cyt2Ba and Cry4Aa toxins differed significantly (F_1,8_ = 11.405, *p* = 0.010). The LC_50_ value for Cry4Aa was estimated at 34.63 ng/mL. The observed LC_50_ value for the binary combination of Cry4Aa+Cyt2Ba was 13.41 ng/mL, whereas the expected LC_50_ value was 61.62 ng/mL, assuming additive action of each of the toxins [31]. These results indicate potentiation in the Cry4Aa+Cyt2Ba protein mixture by a factor of 4.6 (Table 4).

## 3. Discussion

The δ-endotoxins that constitute the major parasporal crystal components of Bti strains (Cry4Aa, Cry4Ba, Cry11Aa and Cyt1Aa) are the best studied of the Bt crystal proteins, both in terms of the insecticidal properties of individual proteins and the interactions among them in the digestive tract of susceptible mosquito species. The present study provides evidence that additional proteins, such as Cry10Aa and Cyt2Ba, which are usually present as minor components in Bti strains, are also important toxicity factors that act in a highly synergistic manner when these proteins are inoculated simultaneously in *A. aegypti* larvae. Cyt2Ba was previously described as a synergy factor for Cry4Aa and in this study we quantified the effects of the interaction on larval mosquito mortality [21].

The insecticidal activity of Cry10Aa in fourth instar larvae of *A. aegypti* was previously estimated at LC_50_ = 2061 ng/mL [17], which is about 7-fold higher than the value that we estimated in second instar larvae of the same species. A decrease in the susceptibility of larvae to infection by pathogens with increasing growth stage is common in insects [32], including their susceptibility to Bt toxins [22,33,34]. 

Several previous studies have described the larvicidal activity of Cyt2Ba protein in mosquito species belonging to the genera *Culex*, *Aedes* and *Anopheles* [19,35,36], although for a given toxin concentration the mortality that was recorded in Cyt2Ba-treated *A. aegypti* larvae was lower than that produced by the Cyt1Aa protein [19]. The estimated 24 h LC_50_ value of Cyt2Ba in second instar larvae of *A. aegypti* obtained in this study was approximately 27-fold lower than the value estimated by others [36]. This may be due to differences in the origin of the mosquito population and history of exposure to Bt toxins, and the fact that Wirth et al. [36] used lyophilized powdered inoculum rather than the freshly-prepared spore + crystal preparations that we employed.

The high potential of Bti proteins against mosquito larvae is mainly attributed to the interactions that occur among the component toxins. Although present at low abundance, Cry10Aa and Cyt2Ba contribute to the insecticidal activity of Bti by potentiation of toxin interactions [5]. Cry10Aa shows synergistic activity with Cyt1Aa [17] and Cry4Aa [37], whereas Cyt2Ba shows synergistic interaction with Cry4Aa [21] and *L. sphaericus* [36] in *A. aegypti*. When large amounts of these proteins were produced in an acrystalliferous Bt strain in the present study, very high levels of toxicity against *A. aegypti* larvae were obtained. 

The LC_50_ value obtained here for the Cry10Aa/Cyt2Ba mixture was about 19 times lower than the value described for the Cry10Aa/Cyt1Aa mixture. This degree of potentiation is one of the highest observed so far for Bti crystal proteins, only comparable to that described for Cyt1A with Cry4Aa and Cry11Aa [38], or Cry4Ba in mixtures with Cyt2Aa2 from Bt *darmstadiensis* against *A. aegypti* larvae [39]. It seems, therefore, that Cyt2 proteins have a greater involvement in toxin synergy than has been attributed to date.

The molecular mechanisms underlying synergistic or other combinatorial effects between Bt insecticidal proteins have been the subject of several studies, although none have focused specifically on Cyt2Ba. The synergistic interaction of Cyt1Aa and Cyt2Aa with Cry4Ba appears to involve binding to the Cry protein through the domain II loops [9,40]. For the interaction between Cyt1Aa and Cry11Aa, specific charged residues have been identified on the Cyt1Aa protein that are involved in binding to Cry11Aa prior to insertion in the midgut epithelial cell membrane [41], although others have proposed that Cyt1Aa is a membrane-bound receptor that uses the exposed charged residues to bind Cry11Aa, thereby facilitating the interaction of the Cry protein with the target cell membrane [42,43]. Oligomerization of the Cyt1Aa toxin is essential for its toxicity in *A. aegypti* [44].

Bti has high larvicidal activity against mosquitoes, although its repeated use can lead to the appearance of resistance. The major components of the crystal, such as Cyt1A, Cry11A, or Cry4 are likely to be the main targets of such resistance. In this study, we demonstrated that Cry10Aa and Cyt2Ba, minor components of the parasporal crystal, have a high mosquitocidal potency and marked synergistic activity when present in a mixture. The optimization of culture conditions that result in improved production of Cry10A and Cyt2Ba may offer a rapid means to produce more effective Bti-based mosquitocidal products.

## 4. Conclusions

The toxicities of Cry10Aa and Cyt2Ba against *A. aegypti* are comparable to the major toxins of Bti and show one of the strongest potentiation effects observed for Bti crystal components to date. This potentiation was much stronger than occurred between Cyt2Ba and Cry4Aa. Further study of the minor crystal components of Bti is likely to provide additional opportunities for the development of safe and effective tools for the biological control of mosquito vectors of medical importance. 

## 5. Materials and Methods 

### 5.1. Bacterial Strains and Plasmids 

*B. thuringiensis* ser. *israelensis* (Bti) was isolated from the commercial insecticide VectoBac-12AS^®^ (Kenogard, Barcelona, Spain). *Escherichia coli* XL1 blue was used for transformation. The recombinant vector pSTAB [45] was used as the protein expression vector, engineered with the gene of interest. The acrystalliferous Bt strain BMB171 was used as the host strain for protein expression [46]. Bt recombinant strains 4Q2-81 pHT606:*cry4Aa* and 4Q2-81 pHT611:*cry4Ba* were kindly provided by Dr. Colin Berry (Cardiff University, Cardiff, UK) [30]. The Bt strains were grown in CCY medium containing 13 mM KH_2_PO_4_, 26 mM K_2_HPO_4_, 10 mL/L Nutrient stock solution (comprising L-glutamine, casein hydrolysate, casitone, yeast extract and glycerol), 1 ml/L metal salts solution [47] at 28 °C with continuous shaking at 200 rpm. All *E. coli* strains were cultured at 37 °C with continuous shaking (200 rpm) in Luria-Bertani (LB) broth (1% tryptone, 0.5% yeast extract, and 1% NaCl, pH 7.0). When required for selective growth, LB medium was supplemented with 20 µg/mL erythromycin (Em) and 100 µg/mL ampicillin (Amp).

### 5.2. Insect Culture

A laboratory colony of *A. aegypti* was started using eggs obtained from Dr. Susana Vilchez, (Universidad de Granada, Granada, Spain). The colony was maintained, under controlled environmental conditions (25 ± 1 °C and 85% RH, and a 16 h:8 h light: dark photoperiod), in the insectary facilities of the Instituto Multidisciplinario de Biología Aplicada (IMBA), Universidad Pública de Navarra, Spain. Adults of both sexes were maintained in BugDorm-1 insect rearing cages (MegaView Science, Taichung, Taiwan) and had continuous access to 20% sucrose solution and intermittent access (3 h/day) to defibrinated horse blood (Thermo Scientific, Waltham, MA, USA) to complete their gonotrophic cycle. Larvae were reared in 250 mL glass beakers (40–50 larvae/beaker) with 100 mL distilled water and brewer’s yeast (1 mg/mL) as food.

### 5.3. Total DNA Extraction and Genomic Sequencing

Genome sequencing was performed to ensure that our Bti clone contained all the expected plasmids and genes, some of which may be lost during laboratory culture. Total genomic DNA (chromosomal + plasmid) was extracted from VectoBac-12AS^®^ strain, following the protocol for DNA isolation from Gram-positive bacteria using the Wizard^®^ Genomic DNA Purification Kit (Promega, Madison, WI, USA). A DNA library was prepared from total DNA and was subsequently sequenced in an Illumina NextSeq500 Sequencer (Genomics Research Hub Laboratory, School of Biosciences, Cardiff University, Cardiff, UK). 

### 5.4. Identification of Cry and Cyt Insecticidal Genes in VectoBac-12AS^®^

Genomic raw sequence data were processed and assembled using CLC Genomics Workbench 10.1.1. Reads were trimmed, filtered by low quality and reads of less than 50 bp were eliminated. Processed reads were assembled de novo using stringent criteria of at least 95 bp overlap and 95% identity. Reads were then mapped back to the contigs for assembly. Genes were predicted using GeneMark [48]. 

To assist in the identification of potential insecticidal proteins, local BLASTP [49] was deployed against a database built in our laboratory comprising the amino acid sequences of known Bt toxins available at http://www.lifesci.sussex.ac.uk/home/Neil_Crickmore/Bt [50], as well as other protein toxins of interest.

### 5.5. Amplification, Cloning and Sequencing of Cyt2Ba, Cry11Aa and Cry10Aa

Primers were designed to amplify the full-length coding sequence of *cyt2Ba, p19-cry11Aa* (including *p19* and *cry11Aa* genes) and the *cry10Aa* operon including *orf1* and *orf2* (Table 5). Primer sequences included XbaI and PstI restriction sites for *cyt2Ba*, as well as SalI and PstI restriction sites for *p19*-*cry11Aa* and SalI and PaeI restriction sites for *cry10Aa*. PCR reactions were performed, from total genomic DNAs, using Phusion DNA polymerase (NEB, Ipswich, UK) and amplicons were gel-purified using NucleoSpin Extract II kit (Macherey-Nagel, Düren, Germany). Purified products were then ligated into pJET1.2/blunt plasmid (CloneJET PCR Cloning Kit, Waltham, MA, USA) following the manufacturer’s instructions. Ligation mixtures were transformed into *E. coli* XL1-Blue using standard procedures [51]. Colony-PCR was applied in order to check positive clones from which plasmid DNA was purified, using the NucleoSpin^R^ plasmid kit (Macherey-Nagel Inc., Bethlehem, PA, USA) following the manufacturer’s instructions. Subsequently, pJET plasmids were verified by sequencing (STABVida, Caparica, Portugal), digested with the appropriate combination of restriction enzymes, electrophoresed in 1% agarose gel and ligated into pre-digested pSTAB vector using the Rapid DNA ligation kit (Thermo Scientific) to obtain the recombinant plasmids pSTAB-*cyt2Ba* and pSTAB-*cry10Aa*. To clone *cry11Aa* the amplicon was ligated in a pSTAB in which *p20* gene was previously introduced, to obtain the recombinant plasmid pSTAB-*p19-cry11Aa-p20*. Ligation products were then electroporated into *E. coli* XL1 blue cells following standard protocols [51]. Positive clones were verified by colony-PCR and plasmids were purified and verified by restriction endonuclease digestion and electrophoresis. Once pSTAB-*cyt2Ba*, pSTAB-*cry10Aa* and pSTAB-*p19-cry11Aa-p20* were obtained, they were introduced into the acrystalliferous Bt strain BMB171. 

*Bacillus* electrocompetent cells were generated as described previously [52]. Briefly, bacteria were grown in 300 mL of Brain heart infusion broth (Pronadisa) at 28 °C under shaking conditions (200 rpm) until the culture reached an OD600 nm of 0.4. Glycine was then added to the culture at 2% and bacterial cells were incubated for another hour, at 28 °C under shaking conditions (200 rpm). Bacterial cells were kept on ice for 5 min, centrifuged at 9000× *g* (4 °C) for 10 min and the pellet was washed three times with F buffer (272 mM sucrose, 0.5 mM MgCl_2_, 0.5 mM K_2_HPO_4_, 0.5 mM KH_2_PO_4,_ pH 7.2). Cells were then resuspended in 600 μL of ice-cold F buffer and stored in aliquots of 50 μL at −80 °C. Plasmids were transformed into the BMB171 strain by electroporation, as described previously [53]. Positive clones were selected by colony-PCR. BMB171 was also transformed with an empty plasmid as a negative control.

### 5.6. Expression of Cyt2Ba, Cry10Aa, Cry4Aa, Cry4Ba and Cry11Aa Recombinant Proteins and SDS-PAGE Analysis

Wild-type Bti and recombinant Bt strains were grown at 28 °C, under shaking conditions (200 rpm), in CCY medium supplemented with 20 µg/mL erythromycin, if required. Crystal formation was observed daily under the optical microscope. After 2–3 days, when ~95% of the cells had lysed, the mixture of spores and crystals was collected by centrifugation at 10,000× *g*, for 10 min at 4 °C. The pellet was washed once with saline solution (1 M NaCl, 10 mM EDTA) and three times with 10 mM KCl. The spore + crystal mixture was finally resuspended in 10 mM KCl and kept at 4 °C until used. Samples of spores and crystals were mixed with 2x sample buffer (Bio-Rad, Hercules, CA, USA), boiled at 100 °C for 5 min, and then subjected to electrophoresis as previously described [54], using Criterion TGX™ 4–20% Precast Gel (Bio-Rad). Gels were stained with Coomassie brilliant blue R-250 (Bio-Rad) and then distained in 30% ethanol and 10% acetic acid. For protein quantification, a 10 µL volume of spore and crystal suspension was solubilized in vitro in 1 mL of alkaline solution (50 mM Na_2_CO_3_, 10 mM DTT, pH 11.3) for 2 h at 37 °C. The protein concentration of each preparation was measured by the Bradford assay (Bio-Rad), using bovine serum albumin (BSA) as a standard. 

### 5.7. Mosquitocidal Activity of the δ-Endotoxins Produced by Bti

The toxicity of Cry10Aa and Cyt2Ba was determined by bioassay against *A. aegypti* second instar larvae. Concentration-mortality bioassays were performed following a modified method described previously [33]. Groups of 10–15 second instar larvae were placed in one well of a 6-well cell culture plate (Costar) and they were exposed to one concentration of Bt (spores+crystals). Each well contained 5 mL of Bt suspension with the corresponding toxin concentration and 0.5 mg of brewer’s yeast as food. Toxin concentrations were 2000, 666, 222, 74, 24.7 and 8.2 ng/mL for Cry10Aa; 4000, 1333, 444, 148, 49.4 and 16.4 ng/mL for Cyt2Ba and 4 × 10^−1^, 2 × 10^−1^, 1 × 10^−1^, 5 × 10^−2^, 2.5 × 10^−2^, 1.2 × 10^−2^ ng/mL for Bti (VectoBac-12AS^®^) as the positive control. Each bioassay was performed at least three times, depending on the toxin. Control insects were mock-infected. Insects were incubated at 25 °C and 16 h:8 h L:D photoperiod. Mortality was recorded at 24 h post-treatment. The concentration-mortality raw data are represented in Table 2 and Table 3. Graphical representation of logit regressions for the individual toxins are summarized in Figure 2. These regressions were used to estimate the median lethal concentration (LC_50_) for the toxins.

To study the synergistic larvicidal activity of Cyt2Ba in binary mixtures with other components of the Bti crystal, a series of preliminary bioassays were made, using a single protein concentration (below 30% mortality). The binary combinations studied, as well as the concentration of proteins used in each case, are shown in Table 1. For those binary combinations that resulted in the highest mortality of inoculated insects, quantitative bioassays were performed in order to determine the potentiation between Cyt2Ba and other toxins. Concentration-mortality bioassays were performed for Cry4Aa at concentrations of 486, 162, 54, 18, 6, 2 ng/mL. Mixtures of Cyt2Ba with either Cry10Aa or Cry4Aa in equal proportions were tested at concentrations of 300, 60, 12, 2.4, 4.8 × 10^−1^ and 9.6 × 10^−2^ ng/mL, and 54, 27, 13.5, 6.74, 3.36 and 1.68 ng/mL, respectively. Each bioassay was performed between five and ten times. In all other aspects, the bioassay procedure and data curation was as described above. Graphical representation of logit regressions for all toxin mixtures are summarized in Figure 2. These regressions were used to estimate the median lethal concentration (LC_50_) for the mixture of the toxins.

### 5.8. Statistical Analysis

Concentration-mortality data were subjected to logit regression to estimate the median lethal concentration (LC_50_) for individual toxins and the mixture of toxins. The significance of treatment and interaction terms was determined by sequential removal of terms from the complete regression model. The observed and expected LC_50_ values for the individual toxins and the toxin mixture in *A. aegypti* were used to evaluate the interaction of Cyt2Ba with Cry10Aa and Cry4Aa. To calculate the expected LC_50_ values for the toxin mixture under the null hypothesis of no interaction the “simple similar action” model was used [31]. This model assumes that concentration-response regression lines for different components of a mixture are parallel and is suitable for testing synergism in chemically similar compounds such as Bt toxins. Because Cyt2Ba and Cry4Aa regression lines are not parallel the synergism factor calculated is only correct for the LC_50_ single point. 

The expected LC_50_ was calculated as follows:

$${\mathrm{LC}}_{50\left(m\right)}={\left[\frac{r_{A}}{{\mathrm{LC}}_{50\left(A\right)}}+\frac{r_{B}}{{\mathrm{LC}}_{50\left(B\right)}}\right]}^{-1}$$

where LC_50(m)_ is the expected LC_50_ of the mixture of toxin A and toxin B, LC_50(A)_ is the observed LC_50_ for toxin A alone, LC_50(B)_ is the observed LC_50_ for toxin B alone and r_A_ and r_B_ represent the relative proportions of toxin A and toxin B in the mixture, respectively. All statistical procedures were performed using R software (v.3.5.1).

## Figures and Tables

**Table 1:** Mortality of *A. aegypti* second instar larvae at 24 h after inoculation with individual Bti δ-endotoxins and the binary combinations Cyt2Ba/Cry10Aa, Cyt2Ba/Cry4Aa, Cyt2Ba/Cry4Ba and Cyt2Ba/Cry11Aa.

Treatment ^1^	Concentration (ng/mL)	Mortality (% ± SD)
Cry10Aa	40	26 ± 5
Cyt2Ba	40	31 ± 8
Cry10Aa+Cyt2Ba (1:1)	80	93 ± 6
Cry4Aa	10	31 ± 10
Cyt2Ba	10	28 ± 23
Cry4Aa+Cyt2Ba (1:1)	20	100 ± 0
Cry4Ba	0.02	32 ± 18
Cyt2Ba	0.02	10 ± 9
Cry4Ba+Cyt2Ba (1:1)	0.04	43 ± 19
Cry11Aa	1.5	40 ± 16
Cyt2Ba	1.5	21 ± 14
Cry11Aa+Cyt2Ba (1:1)	3	46 ± 21

^1^ Control insects experienced no mortality in all cases.

**Table 2:** Mortality of *A. aegypti* second instar larvae at 24 h after inoculation with Cry10Aa, Cyt2Ba and combination of both.

Cry10Aa			Cyt2Ba			Cry10Aa+Cyt2Ba		
ng/mL	Dead/Total	Mortality (% ± SD)	ng/mL	Dead/Total	Mortality (% ± SD)	ng/mL	Dead/Total	Mortality (% ± SD)
2000	39/50	78 ± 2%	4000	84/92	91.3 ± 10%	300	115/124	92.7 ± 9%
666	30/50	60 ± 10%	1333	64/85	75.3 ± 17%	60	86/114	75.4 ± 9%
222	23/41	56.1 ± 2%	444	39/94	41.5 ± 9%	12	75/135	55.6 ± 11%
74	12/44	27.3 ± 12%	148	28/78	35.9 ± 7%	2.4	53/131	40.5 ± 6%
24.7	6/46	13 ± 17%	49.4	22/86	25.6 ± 6%	0.48	34/116	29.3 ± 6%
8.2	1/42	2.4 ± 3%	16.4	21/84	25 ± 13%	0.096	23/120	19.2 ± 14%

Control insects experienced no mortality in all cases.

**Table 3:** Mortality of *A. aegypti* second instar larvae at 24 h after inoculation with Cry4Aa, Cyt2Ba and combination of both.

Cry4Aa			Cyt2Ba			Cry4Aa+Cyt2Ba		
ng/mL	Dead/Total	Mortality (% ± SD)	ng/mL	Dead/Total	Mortality (% ± SD)	ng/mL	Dead/Total	Mortality (% ± SD)
486	63/70	90 ± 11%	4000	84/92	91.3 ± 10%	54	139/163	85.3 ± 13%
162	53/71	74.6 ± 17%	1333	64/85	75.3 ± 17%	27	111/155	71.6 ± 17%
54	42/70	60 ± 13%	444	39/94	41.5 ± 9%	13.5	87/200	43.5 ± 14%
18	27/73	37 ± 11%	148	28/78	35.9 ± 7%	6.74	58/142	40.8 ± 24%
6	13/64	20.3 ± 10%	49.4	22/86	25.6 ± 6%	3.36	11/102	10.8 ± 16%
2	7/71	9.9 ± 7%	16.4	21/84	25 ± 13%	1.68	5/73	6.8 ± 8%

Control insects experienced no mortality in all cases.

**Table 4:** Logit regression of concentration-mortality results of wild-type (VectoBac-12AS^®^) and recombinant proteins and their mixtures in *A. aegypti* second instar larvae at 24 h.

Treatment ^(a)^	Regression		LC_50_ Observed	FL (95%) ^(b)^		LC_50_ Expected ^(c)^			FL (95%) ^(b)^	
	Slope ± SE	Intercept ± SE	(ng/mL)	Lower	Upper	(ng/mL)	Synergistic Factor ^(d)^	Potency	Lower	Upper
Cyt2Ba	0.59 ± 0.13	−3.33 ± 0.79	279.37	190.20	410.38	-	-	1	-	-
Cry10Aa	0.74 ± 0.09	−4.26 ± 0.53	299.62	245.06	366.34	-	-	0.93	0.78	1.12
Cry4Aa	0.78 ± 0.02	−2.77 ± 0.09	34.63	29.73	40.34	-	-	8.07	6.40	10.17
Cry10Aa+Cyt2Ba ^(e)^	0.45 ± 0.05	−0.64 ± 0.14	4.22	3.25	5.50	289.27	68.55	66.20	58.52	74.61
Cry4Aa+Cyt2Ba ^(e)^	1.22 ± 0.17	−3.17 ± 0.46	13.41	12.55	14.33	61.62	4.60	20.83	15.15	28.63
VectoBac-12AS^®^	1.51 ± 0.29	3.44 ± 0.73	1.02 × 10^−1^	9.34 × 10^−2^	1.11 × 10^−1^	-	-	2.73 × 10^3^	2.03 × 10^3^	3.69 × 10^3^

^(a)^ Inocula comprised spore+crystal mixtures. Control insects experienced no mortality in all cases. ^(b)^ FL: Fiducial limits (95%). ^(c)^ Expected LC_50_ calculated by the method of Tabashnik (1992). ^(d)^ Synergism factor defined as the ratio of the expected LC_50_ and the observed LC_50_. ^(e)^ Toxins were present in equal amounts in the experimental inocula.

**Table 5:** Sequences of PCR and sequencing primers.

Primer Name	Primer Sequence	Reference
Cyt2B-Fw-XbaI	5′-TTCTAGAGATAATGAAGGAGGGGAGTC-3′	This study
Cyt2B-Rv-PstI	5′-CCTGCAGCAAAATTAAATTGCTGAGTTACTATAATAAC-3′	This study
Cry10A-Fw-SalI	5′-ATGTCGACTTGCAACAGAAAAGAGTTGTGTC-3′	[17]
Cry10A-Rv-PaeI	5′-GAGCATGCACATTTCCCCACAATTTTCA-3′	[17]
Cry10A-test-Fw	5′-CGAAATTGTCAGACATAGAGAG-3′	This study
Cry10A-test-Rv	5′-GAATTACCAAGTCTCCACCTG-3′	This study
p20-Fw-PstI	5′-CCTGCAGGGATAAAATTGGAGGATAATTGATG-3′	This study
p20-Rv-PaeI	5′-GGCATGCGTTTCCAGTGCATTCAATTTAC-3′	This study
p19-Fw-SalI	5′-GTGTCGACGTTTTTTAAAATTGCATAGAAGGG-3′	This study
Cry11A-Rv-PstI	5′-CTCTGCAGGTGCTAACATGACTTCTACTTTAG-3′	This study
Cry11A-test	5′-GGTCATAATTTATGAATAAAAATATGAC-3′	This study

Restriction enzyme sites are underlined.
